# The Ability of Heart Failure Specialists to Accurately Predict NT-proBNP Levels Based on Clinical Assessment and a Previous NT-proBNP Measurement

**DOI:** 10.2174/1874192400802010036

**Published:** 2008-06-05

**Authors:** Tara L Sedlak, Mann Chandavimol, Anna Calleja, Catherine Clark, Margaret Edmonds, Aihua Pu, Karin H Humphries, Andrew Ignaszewski

**Affiliations:** 1 University of British Columbia; 2 University of British Columbia; 3 University of British Columbia; 4 St. Paul’s Hospital, Vancouver; 5 St. Paul’s Hospital, Vancouver; 6 Centre for Health Evaluation & Outcome Sciences; 7 University of British Columbia, Centre for Health Evaluation & Outcome Sciences; 8 University of British Columbia

## Abstract

**Background:**

The value of routine aminoterminal pro type B natriuretic peptide (NT-proBNP) measurements in outpatient clinics remains unknown.

**Objectives:**

We sought to determine the accuracy with which heart failure (HF) specialists can predict NT-proBNP levels in HF outpatients based on clinical assessment.

**Methods:**

We prospectively studied 160 consecutive HF patients followed in an outpatient multidisciplinary HF clinic. During a regular office visit, HF specialists were asked to estimate a patient’s current NT-proBNP level based upon their clinical assessment and all available information from their chart, including a previous NT-proBNP level (if available). NT-proBNP estimations were grouped into prognostic categories (<125, 125-1000, 1000-4998, or ≥4999 pg/mL) and comparisons made between actual and estimate values.

**Results:**

Overall, HF specialists estimated 67.5% of NT-proBNP levels correctly. After adjusting for clinical characteristics, knowledge of a prior NT-proBNP measurement was the only significant predictor of estimation accuracy (p=0.01). Compared to patients with a prior NT-proBNP level <125 pg/mL, physicians were 95% less likely to get a correct estimation in patients with the highest prior NT-proBNP level (≥4999 pg/mL).

**Conclusion:**

HF specialists are reasonably accurate at estimating current NT-proBNP levels based upon clinical assessment and a previous NT-proBNP level, if those levels were < 4999 pg/mL. Likely, initial but not routine NT-proBNP measurements are useful in outpatient HF clinics.

## INTRODUCTION

B-type natriuretic peptide (BNP) is released from ventricular myocardial tissue in response to increased wall stress. Both BNP and aminoterminal pro-type B natriuretic peptide (NT-proBNP), an inactive amino acid product of BNP prohormone cleavage, can be measured in the serum and both are typically elevated in heart failure (HF).

NT-proBNP is frequently measured at regular intervals in HF outpatients. Multiple studies have demonstrated the value of NT-proBNP in the diagnosis of congestive HF [1-6]. Recent research suggests that serial NT-proBNP measurements may also have a role in prognostic evaluation [7-2] and therapy optimization [13-16]. To date, however, studies have not been conducted looking at the cost-effectiveness of regular outpatient NT-proBNP measurements. Further, in the 2007 Canadian Cardiovascular Society Consensus Conference recommendations on HF, sequential measurements of 

BNP/NT-proBNP levels were given a class IIb recommendation [17].

Skepticism remains regarding the value of routine NT-proBNP measurements in outpatient clinics. Specifically, it is unknown whether clinicians are able to accurately predict NT-proBNP levels on an outpatient basis. Our primary objective was to determine the accuracy with which HF specialists can predict NT-proBNP levels in HF patients based on clinical assessment during an office visit. Our secondary objective was to determine whether certain clinical characteristics (i.e., gender, BMI, age, eGFR, NYHA, and the absence of a previous NT-proBNP level) were predictors of accurate NT-proBNP estimations.

## METHODS

### Patient Characteristics and Study Protocol

We enrolled 160 consecutive patients >18 years with known HF attending the outpatient Heart Function, Post-Heart Transplant, and Maintenance Clinics at St. Paul's Hospital in Vancouver, British Columbia, Canada. Ethics approval for the study was obtained from the University of British Columbia/Providence Health Care Research Ethics Board. NT-proBNP levels were drawn from each HF patient upon arrival to clinic. Each patient was then assessed by one of five HF specialists as a regular office visit. All HF specialists were cardiologists trained specifically in HF and all patients were seen in St. Paul’s Hospital, a high volume tertiary care center. During this visit, physicians were asked to estimate the patient's current NT-proBNP level based upon their clinical assessment and all available information from the patient's chart, including a previous NT-proBNP level (if available). Actual and estimated NT-proBNP levels were then recorded by an independent physician, in addition to gender, age, previous NT-proBNP level (if available), current body mass index (BMI), most recent glomerular filtration rate (eGFR), and current New York Heart Association (NYHA) class. NT-proBNP levels were grouped into prognostic categories (<125, 125-1000, 1000-4998 or ≥4999 pg/mL) outlined by a recent study by Rothenburger *et al*. [10]. If the estimated and the actual NT-proBNP prognostic categories were the same, we considered this as an accurate estimation.

### NT-proBNP Measurement

NT-proBNP levels were measured by the Elecsys® proBNP immunoassay on the Roche Elecsys® 1010 immunoassay analyzer.

### Statistical Analyses

For univariate analysis, the Chi-square test was used to examine the association between correct physician NT-proBNP estimation and the following factors: gender, BMI <25 kg/m2, age <65, eGFR>60 mL/min./1.73m2, NYHA class I or II, the absence of a previous NT-proBNP measurement, and the category of the most recent prior NT-proBNP level (if available). For multivariable analysis, a logistic regression model was used to investigate the association between the prior NT-proBNP prognostic category and correct physician NT-proBNP estimation, adjusted for gender, BMI, age, eGFR, NYHA, and the time difference between the current test and prior test.

## RESULTS

Baseline characteristics of the patients are displayed in Table **1**. In general, more patients were male (69.5%), less than 65 years of age (56.1%), had an elevated BMI ≥ 25 (76.2%), were NYHA I or II (76.4%), and had a previous NT-proBNP (82.3%). Most patients with a previous NT-proBNP level had a measurement between 1000-4998 pg/mL and a time interval between measurements of 6 to 12 months.

Overall, HF specialists estimated 67.5% of NT-proBNP levels correctly. In univariate analysis, a prior NT-proBNP measurement was significantly associated with estimation accuracy (p=0.01). Importantly, the level of prior NT-proBNP measurement was significantly associated with estimation accuracy (p=0.002). For patients with a prior NT-proBNP test, physician estimations were good for patients with a prior BNP level <125 pg/mL (92.3%) and BNP levels between 125-999 pg/mL (84.2%), but the accuracy reduced greatly as the prior NT-proBNP levels increased. The correct estimation was only 66.7% in patients with a prior BNP level between 1000 and 4998 pg/mL. The accuracy of physician estimations for patients with a prior NT-proBNP level ≥4999 further reduced to 47.1%, which was similar to that of patients without a prior NT-proBNP test (46.2%). No significant association was observed for any other factors.

The results of multivariable analysis echoed that of the univariate analysis. The absence of a prior NT-proBNP measurement significantly reduced the accuracy of physician estimation (OR: 0.05; 95%CI 0.004-0.49) (Table **2**). The level of prior NT-proBNP also greatly affected the estimation accuracy. Compared to patients with a prior NT-proBNP level <125 pg/mL, physicians were 95% less likely to get a correct estimation, in patients with the highest prior NT-proBNP level (≥4999 pg/mL). This was after adjustment for gender, BMI, age, GFR, NYHA, and the time difference between the current and prior test.

## DISCUSSION

We found that HF specialists are relatively accurate at estimating NT-proBNP prognostic categories based upon clinical assessment and a previous NT-proBNP level. They are less accurate at predicting NT-proBNP levels in new patients with no prior NT-proBNP measurement and in patients with a prior NT-proBNP level ≥4999 pg/mL. Renal insufficiency, NYHA class, BMI, gender, and age do not appear to be predictive markers of accurate NT-proBNP estimations. To our knowledge, we are the first group to establish that perhaps initial but not routine NT-proBNP measurements may be useful in outpatients whose initial measurements were < 4999 pg/mL.

### NT-proBNP/BNP in the Diagnosis, Prognostic Evaluation, and Management of Patients with CHF

To date, NT-proBNP/BNP has been studied in a variety of clinical settings. In the diagnosis of congestive HF, the use of NT-proBNP and BNP measurement in the emergency department has been well established [5,6]. In contrast, the use of neurohormones in the outpatient diagnosis of HF has been less well studied. In a study by Groenning *et al*., NT-proBNP measurement in the outpatient setting identified patients with symptoms of HF and a low left ventricular ejection fraction (LVEF) of less than 40% with a positive predictive value of 0.11 and a negative predictive value of 1.00 [4]. In a study by Wright *et al*. that recruited patients presenting to their general practitioner with dyspnea and/or edema, knowledge of the NT-proBNP level improved the diagnostic accuracy of heart failure from 8% (control group) to 21% (NT-proBNP group) (p=0.002)[3]. Finally, Zaphiriou *et al*. performed a study on 306 patients referred to a heart failure clinic by their general practitioners with suspected heart failure. They found that NT-proBNP and BNP were good rule-out tests with high negative predictive values (0.97 and 0.87, respectively) but low positive predictive values (0.44 and 0.59, respectively)[2].

Recent research suggests that serial NT-proBNP measurements may have a role in prognostic evaluation [7-2] and therapy optimization [13-16]. In prognostic evaluation, NT-proBNP/BNP has been found to be an independent predictor of sudden death [7], HF hospital admissions [4], and depressed left ventricular function [7]. In a recent systematic review of 19 studies using BNP to estimate the relative risk of death or cardiovascular events in HF patients, BNP was found to be a strong prognostic indicator [18]. Each 100 pg/mL increase in BNP was associated with a 35% increase in the relative risk of death. Further, Maisel *et al*. in the Rapid Emergency Department Heart Failure Outpatient Trial (REDHOT) study found that BNP is a potent predictor of the 90-day combined event rate of congestive HF visits or admissions and mortality in patients admitted from the emergency department with acute HF [19]. In the Valsartan Heart Failure Trial, HF patients with an initial BNP level greater than 97 pg/mL were 2.1 times more likely to die at 12 months [20]. As well, in the Australia/New Zealand Heart Failure Study, BNP was superior to LVEF in predicting mortality and HF in patients with ischemic LV dysfunction [9]. Above-median BNP levels conferred a 3-fold-increased risk of mortality. In pre-heart transplant assessment [10], an arbitrary cutoff point of >2000 pg/mL for patients with dilated cardiomyopathy and >1000 pg/mL for patients with LV dysfunction secondary to coronary artery disease included 90% of all patients who were clinically considered for cardiac transplantation. As well, BNP appears to be useful in predicting the progression of HF after cardiac resynchronization therapy (CRT) [21]. Specifically, patients with high BNP values after 1 month of CRT have worse prognosis during follow-up.

Finally, in therapy optimization, Troughton *et al*. demonstrated that titration of therapy guided by NT-proBNP levels in symptomatic HF patients decreased hospital re-admission rates and cardiac mortality at 9.5 months follow-up, as compared to conventional follow up [14]. BNP may guide the introduction of medications in HF. Richards *et al*. demonstrated that carvedilol reduced mortality rates in HF to a greater extent in patients with higher pretreatment BNP levels (9). More recently, in the Systolic Heart Failure Treatment Supported By BNP (STARS-BNP) Multicenter Randomized Trial patients randomized to the BNP guided treatment group suffered fewer events (death or hospitalizations) for HF than in the control group (p<0.001) [16].

The above studies suggest that knowledge of an initial NT-proBNP and perhaps repeat NT-proBNP levels may guide diagnosis, prognosis and therapy management. The most recent HF guidelines, specifically, the 2007 Canadian Cardiovascular Society Consensus Conference Recommendations on Heart Failure, support the initial measurement of NT-proBNP/BNP for diagnosis (class I recommendation) and prognostic stratification (class IIa recommendation). The guidelines do not however support sequential measurements of BNP/NT-proBNP levels in the outpatient setting (class IIb recommendation) [17]. To date, no study has demonstrated that routine measurement of NT-proBNP levels in the outpatient setting is cost-effective. In our study, HF specialists were relatively accurate at estimating NT-proBNP levels when a prior NT-proBNP measurement was available, irrespective of the time interval between measurements. In contrast, they were less accurate at predicting NT-proBNP levels in new patients with no prior NT-proBNP level and in patients with patients with a prior NT-proBNP level ≥4999 pg/mL.

### Predictors of NT-proBNP Estimation Accuracy

Recent studies suggest that NT-proBNP levels are altered in overweight individuals. Specifically, NT-proBNP levels are significantly lower in overweight and obese individuals with HF compared with lean patients [22,23]. Age and gender alter NT-proBNP levels. Redfield *et al*. found in a recent study that BNP increased significantly with age and was higher in women than men [24]. Renal dysfunction also alters NT-proBNP levels in that eGFR and BNP are inversely related [25].

We hypothesized that age>65 years, female gender, elevated BMI (>25 kg/m^2^), renal dysfunction (eGFR <60mL/min./1.73m^2^), NYHA class I or II, and absence of a prior NT-proBNP level would be predictive of NT-proBNP estimation accuracy. Surprisingly, we found that a prior NT-proBNP measurement was the only factor significantly associated with the accuracy of physician estimation. Age >65 years, female gender, elevated BMI (>25 kg/m^2^), renal dysfunction (eGFR <60mL/min./1.73m^2^), NYHA class I or II were not associated with accurate NT-proBNP estimations. In our study, physicians had access to all patient records in the heart failure clinic, including GFR, BMI, etc. We believe that HF specialists purposefully altered their NT-proBNP estimation as a result of this knowledge enabling them to guess correctly.

Interestingly, in patients with a prior NT-proBNP level, accuracy reduced greatly as the prior NT-proBNP levels increased. Compared to patients with a prior NT-proBNP level <125 pg/mL, physicians were 95% less likely to get a correct estimation, in patients with the highest prior NT-proBNP level (≥4999 pg/mL). Karabulut *et al*. found that while NT-proBNP levels increase significantly with each increasing class of disease, there is a wider range of NT-proBNP levels in NYHA classes III and IV [26]. This may account for our relative inaccuracy at estimating NT-proBNP levels in higher NYHA classes. In these patients, it may be useful to measure NT-proBNP levels on a more routine basis.

## CONCLUSIONS

In conclusion, we found that heart failure specialists are fairly accurate at predicting NT-proBNP levels in heart failure patients only if a prior NT-proBNP measurement is available and the prior level was < 4999 pg/mL. In these patients, likely, initial but not routine measurements of NT-proBNP are useful in outpatient heart failure clinics.

## Figures and Tables

**Table 1.** Baseline Characteristics of HF Patients

**Characteristic**	**n**	** %**
Female	50	30.5
BMI≥ 25 kg/m^2^	125	76.2
Age≥ 65 years	72	43.9
eGFR≥60 mL/min./1.73m^2^	85	51.8
NYHA III/IV	38	23.6
Prior NT-proBNP measurement	135	82.3
Time from last measurement		
<6month	39	28.9
6 month to 12 month	61	45.2
≥12 month	35	25.9
Prior NT-proBNP level (pg/mL)		
<125	13	9.6
125-1000	42	31.1
1000-4999	63	46.7
≥4999	17	12.6

BMI = body mass index; eGFR = glomerular filtration rate; NYHA = New York Heart Association; NT-proBNP = aminoterminal pro type B natriuretic peptide.

**Table 2.** Adjusted Odds Ratios of Correct Estimation of Current NT-proBNP Category

**Factors**	**Odds Ratio**	**95% CI**	**p-value**
No prior NT-proBNP measurement	0.05	(0.004,0.49)	0.01
Time from last measurement			
≥ 12 months	1.00		
6-12 months	0.67	(0.219,2.062)	0.48
<6 months	0.57	(0.177,1.851)	0.35
Prior NT-proBNP level (pg/mL)			
<125	1.00		
125-999	0.15	(0.016,1.377)	0.09
1000-4998	0.48	(0.049,4.686)	0.53
≥4999	0.05	(0.004,0.605)	0.02
Female	0.82	(0.364,1.864)	0.64
BMI ≥25 kg/m^2^	1.16	(0.456,2.958)	0.75
Age ≥ 65	1.03	(0.424,2.515)	0.94
eGFR ≥ 60 mL/min./1.73m^2^	0.53	(0.191,1.483)	0.23
NYHA class III/IV	0.69	(0.288,1.657)	0.41

BMI = body mass index; eGFR = glomerular filtration rate; NYHA = New York Heart Association; NT-proBNP = aminoterminal pro type B natriuretic peptide.

## ABBREVIATIONS

Congestive heart failure

Aminoterminal pro type B natriuretic peptide
